# Update on the diagnosis and treatment of neuromyelits optica spectrum disorders (NMOSD) – revised recommendations of the Neuromyelitis Optica Study Group (NEMOS). Part I: Diagnosis and differential diagnosis

**DOI:** 10.1007/s00415-023-11634-0

**Published:** 2023-04-06

**Authors:** Sven Jarius, Orhan Aktas, Ilya Ayzenberg, Judith Bellmann-Strobl, Achim Berthele, Katrin Giglhuber, Vivien Häußler, Joachim Havla, Kerstin Hellwig, Martin W. Hümmert, Ingo Kleiter, Luisa Klotz, Markus Krumbholz, Tania Kümpfel, Friedemann Paul, Marius Ringelstein, Klemens Ruprecht, Makbule Senel, Jan-Patrick Stellmann, Florian Then Bergh, Hayrettin Tumani, Brigitte Wildemann, Corinna Trebst, Neuromyelitis Optica Study Group (NEMOS)

**Affiliations:** 1 Molecular Neuroimmunology Group, Department of Neurology, University of Heidelberg, Heidelberg, Germany; 2 Department of Neurology, Medical Faculty, Heinrich Heine University Düsseldorf, Düsseldorf, Germany; 3 Department of Neurology, St. Josef Hospital, Ruhr University Bochum, Bochum, Germany; 4 Department of Neurology, Charité-Universitätsmedizin Berlin, corporate member of Freie Universität Berlin and Humboldt-Universität zu Berlin, Berlin, Germany; 5 Experimental and Clinical Research Center, a Cooperation between the Max Delbrück Center for Molecular Medicine in the Helmholtz Association and Charité-Universitätsmedizin Berlin, Berlin, Germany; 6 Max Delbrück Center for Molecular Medicine in the Helmholtz Association (MDC), Berlin, Germany; 7 Department of Neurology, School of Medicine, Technical University Munich, Klinikum rechts der Isar, Munich, Germany; 8 Department of Neurology and Institute of Neuroimmunology and MS (INIMS), University Medical Center Hamburg-Eppendorf, Hamburg, Germany; 9 Institute of Clinical Neuroimmunology, LMU Hospital, Ludwig-Maximilians-Universität München, Munich, Germany; 10 Data Integration for Future Medicine (DIFUTURE) Consortium, Ludwig-Maximilians-Universität München, Munich, Germany; 11 Department of Neurology, Hannover Medical School, Hannover, Germany; 12 Marianne-Strauß-Klinik, Behandlungszentrum Kempfenhausen für Multiple Sklerose Kranke, Berg, Germany; 13 Department of Neurology with Institute of Translational Neurology, University of Münster, Münster, Germany; 14 Department of Neurology and Pain Treatment, Immanuel Klinik Rüdersdorf, University Hospital of the Brandenburg Medical School Theodor Fontane, Rüdersdorf bei Berlin, Germany; 15 Faculty of Health Sciences Brandenburg, Brandenburg Medical School Theodor Fontane, Rüdersdorf bei Berlin, Germany; 16 Department of Neurology and Stroke, University Hospital of Tübingen, Tübingen, Germany; 17 NeuroCure Clinical Research Center, Charité Universitätsmedizin Berlin, corporate member of Freie Universität Berlin and Humboldt-Universität zu Berlin, and Berlin Institute of Health, and Max Delbrück Center for Molecular Medicine, Berlin, Germany; 18 Department of Neurology, Center for Neurology and Neuropsychiatry, LVR-Klinikum, Heinrich Heine University Düsseldorf, Düsseldorf, Germany; 19 Department of Neurology, University of Ulm, Ulm, Germany; 20 APHM, Hopital de la Timone, CEMEREM, Marseille, France; 21 Aix Marseille Univ, CNRS, CRMBM, Marseille, France; 22 Department of Neurology, University of Leipzig, Leipzig, Germany

**Keywords:** Neuromyelitis optica spectrum disorders (NMOSD), Neuromyelitis optica (NMO), Optic neuritis, Myelitis, Diagnostic criteria, Diagnosis, Differential diagnosis, MOG antibody-associated encephalomyelitis (MOG-EM), MOG antibody-associated disease (MOGAD), Magnetic resonance imaging (MRI), Serology, Cerebrospinal fluid (CSF), Optic coherence tomography (OCT), Clinical presentation, Aquaporin-4 (AQP4), Myelin oligodendrocyte glycoprotein (MOG), Autoantibodies

## Abstract

The term ‘neuromyelitis optica spectrum disorders’ (NMOSD) is used as an umbrella term that refers to aquaporin-4 immunoglobulin G (AQP4-IgG)-positive neuromyelitis optica (NMO) and its *formes frustes* and to a number of closely related clinical syndromes without AQP4-IgG. NMOSD were originally considered subvariants of multiple sclerosis (MS) but are now widely recognized as disorders in their own right that are distinct from MS with regard to immunopathogenesis, clinical presentation, optimum treatment, and prognosis. In part 1 of this two-part article series, which ties in with our 2014 recommendations, the neuromyelitis optica study group (NEMOS) gives updated recommendations on the diagnosis and differential diagnosis of NMOSD. A key focus is on differentiating NMOSD from MS and from myelin oligodendrocyte glycoprotein antibody-associated encephalomyelitis (MOG-EM; also termed MOG antibody-associated disease, MOGAD), which shares significant similarity with NMOSD with regard to clinical and, partly, radiological presentation, but is a pathogenetically distinct disease. In part 2, we provide updated recommendations on the treatment of NMOSD, covering all newly approved drugs as well as established treatment options.

**Supplementary Information:**

The online version contains supplementary material available at 10.1007/s00415-023-11634-0.

## Introduction

The term ‘neuromyelitis optica spectrum disorders’ (NMOSD) is used to refer to inflammatory disorders of the central nervous system (CNS) that predominantly affect the optic nerves, the spinal cord, and, less frequently, the brainstem, causing acute attacks of optic neuritis, myelitis, and brainstem encephalitis [92, 93, 228]. In the majority of cases, NMOSD is associated with pathogenic immunoglobulin G (IgG) antibodies to aquaporin-4 (AQP4), the most common water channel in the CNS [89, 124, 125]. The discovery of AQP4-IgG in 2004/2005 had significant implications for the nosology, diagnosis, and treatment of NMOSD [228, 230]. The neuromyelitis optica study group (NEMOS; see www.nemos-net.de), founded in 2008 as a nation-wide network of primary, secondary, and tertiary care centers, seeks to broaden the understanding of NMOSD and clinically related (but pathogenetically distinct) disorders, such as myelin oligodendrocyte glycoprotein antibody-associated encephalomyelitis (MOG-EM; also termed MOG antibody-associated disease, MOGAD) [77, 134]. The group has published several national and international multicenter investigations and practical recommendations on the diagnosis and treatment of NMOSD and MOG-EM/MOGAD [6, 9, 10, 16, 59, 75, 76, 83, 84, 86, 87, 112–115, 154, 177, 178, 180, 200, 212, 213]. Tying in with the recommendations published by the group in 2014 [213], this two-part article series gives updated recommendations on the diagnosis (part 1) and therapy (part 2) of NMOSD.

## Methods

A core working group consisting of representatives of 12 German neurological university centers (Charité—Universitätsmedizin Berlin, Ruhr University Bochum, Heinrich-Heine University Düsseldorf, University of Hamburg, Hannover Medical School, University of Heidelberg, University of Leipzig, Ludwig Maximilian University Munich, Technical University of Munich, University of Münster, University of Tübingen, University of Ulm), all of which are members of NEMOS, wrote a first draft, based on expert discussion during NEMOS meetings, with the Heidelberg and Hannover centers jointly responsible. The draft was then revised in several rounds based on expert comments and circulated to 121 study group members, representing 32 university and 30 non-university neurological institutions, for further comments and final approval (see Acknowledgements for a list of all study group members). Further revisions were made by the core working group following peer review, and the final manuscript was again sent to all study group members for comments and final approval.

## When to consider NMOSD?—Hints from epidemiology

NMOSD must be considered as a potential differential diagnosis in all patients presenting with CNS inflammation of putative autoimmune etiology, especially if they have optic neuritis (ON), myelitis, or brainstem encephalitis, irrespective of sex, age, and ethnicity.

Significant heterogeneity exists among epidemiological studies with regard to inclusion criteria and methodology; in particular, not all studies have differentiated between AQP4-IgG-positive and AQP4-IgG-negative patients. However, some risk factors have been identified. NMOSD has been shown to predominantly affect females, to be more common in some non-Caucasian populations, and to start in adulthood in the majority of cases.

The incidence showed a peak in middle-aged adults in most studies (e.g., ~ 40 years among AQP4-IgG-positive and 38.5 years among AQP4-IgG-negative patients in a large European cohort [87]). However, a diagnosis of NMOSD must be given serious consideration also in the elderly and in children. Late-onset (LO) NMOSD (> 60 years) accounted for 20–28% of all incident NMOSD cases in some (mixed AQP4-IgG-positive and AQP4-IgG-negative) cohorts [158], and the disease may even start only at very old age (> 75 years). LO-NMOSD has been associated with a less favorable prognosis [28, 119, 189, 191]. This compares to a median age at clinical disease onset of around ~ 30 years in multiple sclerosis (MS) and adult MOG-EM/MOGAD.

While NMOSD must be considered in both sexes, there is a strong female preponderance irrespective of ethnicity, with a female:male ratio of up to 10:1 among AQP4-IgG-seropositive patients, who account for the vast majority of NMOSD, and up to 3:1 in seronegative patients [16, 84, 87]. Among AQP4-IgG-positive patients of fertile age, the sex ratio may even exceed 20:1 [16]. Accordingly, female patients with NMOSD are significantly more likely to be AQP4-IgG-positive than male patients [16]. In contrast, MOG-EM/MOGAD affects women and men in almost equal measure. MS shows a female preponderance similar to that reported for seronegative NMOSD.

Although a diagnosis of NMOSD must be taken into account irrespective of ethnic origin, the overall incidence and prevalence of NMOSD is thought to be higher among non-whites than among whites [19, 39], with the highest rates found for patients of African descent in some studies [39, 158]. This is in contrast to MS, which is most common in Caucasians of northern European ancestry. Reported incidence and prevalence estimates for NMOSD vary substantially among studies from different regions of the world (0.28–0.73/100,000 person-years and 0.52–10/100,000 persons, respectively) [56, 82, 143, 158], suggesting differences in genetic background and environmental factors across the populations analyzed [39, 220]. However, differences in study methodology and inclusion criteria may play a role as well. The few studies that reported antibody-specific incidences all found a much higher incidence of AQP4-IgG-positive than AQP4-IgG-negative NMOSD [156, 157, 190]; in line with this, AQP4-IgG-positive patients accounted for most cases of NMO or NMOSD in major cross-sectional studies.

The proportion of patients with NMOSD among patients with inflammatory demyelinating disorders (IDD) varies among populations. While NMOSD accounts only for a small proportion of white adult patients with IDD, most of whom have classic MS, rates are much higher in some (but not all [188]) Asian populations [19, 142, 208, 210, 220], at least in part reflecting the well-known lower prevalence of MS in Asia [147, 210]. NMOSD was initially thought to be more common than MOG-EM/MOGAD among adult patients with IDD [55, 106] and less frequent than MOG-EM/MOGAD among children with IDD [15, 34, 188]; however, recent data suggest that cases of MOG-EM/MOGAD may outnumber those of both AQP4-IgG-positive and AQP4-IgG-negative NMOSD also in adults, in both Asian and non-Asian populations [121, 188]. This change may possibly result from the recent rise in awareness regarding MOG-EM/MOGAD and the increasing availability of serological tests for MOG-IgG.

Certain human leukocyte antigen (HLA) types are associated with an increased risk of developing AQP4-IgG-positive NMOSD (e.g., HLA-DRB1*03 in Europe, Brazil, and India [odds ratio 1.79–9.23] [2]; HLA-DPB1*0501 in southern Han Chinese; HLA-DPB1*0501 and HLA-DRB1*1602 in Japanese; and HLA- DRB1*04:04 and HLA- DRB1*10:01 in Muslim Arab Israelis) [82]. However, HLA typing is not required to make a diagnosis of NMOSD and is currently not recommended outside the context of scientific studies. Other risk factors are less well established (e.g., vitamin D deficiency, smoking, low number of infections in early life) [82]. In contrast to MS, no convincing evidence for an increase in incidence with increasing latitude has been observed in NMOSD [19, 208, 210, 232].

## When to consider NMOSD?—Clinical characteristics

NMOSD must be considered as a differential diagnosis in all patients presenting with acute attacks of transverse myelitis and/or (unilateral or, less frequently, bilateral) acute ON, the two clinical hallmarks of NMOSD, but also in patients presenting with suspected encephalitis or brainstem encephalitis. Myelitis and ON may occur either simultaneously or, far more often, successively. The combination of myelitis and ON gave the disease its name [82, 87].

Patients with suspected myelitis must be examined for sensory, motor (including respiratory), bladder/bowel, and sexual dysfunction. Moreover, pain should be assessed, ideally using structured questionnaires in addition to clinical examination. Pain, as an immediate or long-term sequela of myelitis, is highly prevalent in both AQP4-IgG-positive and AQP4-IgG-negative NMOSD [8, 9, 17, 104, 162] and includes neuropathic pain [9], painful tonic spasms [22, 104, 129], neuropathic pruritus [35, 54], and spasticity-associated pain [9]; dysesthesia (often girdle-like in the case of thoracic myelitis) and hyperalgesia/allodynia are frequent [162].

ON mostly leads to blurred vision, decline in visual acuity, headache, retrobulbar pain/pain upon eye movement, phosphenes, a relative afferent pupillary defect (if unilateral or asymmetrical), i.e., a positive Marcus-Gunn sign/swinging flashlight test, sometimes optic disk swelling/papilledema visible upon fundoscopy (a finding much more frequent in MOG-EM/MOGAD), visual field deficiency, and/or color desaturation [165]. Both eyes must be examined, since both optic nerves may be affected simultaneously with only minor or even subclinical damage possible in one eye. However, bilateral ON, which is rare in MS, is possibly more common in MOG-EM/MOGAD than in NMOSD, at least at onset [84, 87, 170].

Extra-opticospinal symptoms must not lead to premature exclusion of NMOSD. Following the discovery of AQP4-IgG, the clinical spectrum was found to be much broader than initially thought and to include, although more rarely, supratentorial and brainstem encephalitis. The latter often affects the dorsal medulla oblongata, causing intractable acute or subacute nausea, vomiting and/or hiccups (either in combination or isolated; constant or episodic), the so-called area postrema syndrome (APS) [197]. APS is much less common in AQP4-IgG-negative patients with NMOSD [87] and in MOG-EM/MOGAD [62, 84]. The diagnosis should be made according to the criteria proposed by Shosha et al. [197]. These require persistence of symptoms for at least 48 h if magnetic resonance imaging (MRI) shows no evidence of acute area postrema involvement (considering both the rarity of APS as a cause of vomiting and hiccups in the general population and the broad spectrum of differential diagnoses), whereas shorter duration is deemed sufficient if MRI shows such involvement. Moreover, demonstration of a lack of complete resolution after symptomatic therapy (antiemetics, IV fluid, hiccup treatments) and exclusion of other etiologies (gastrointestinal/mediastinal, metabolic [e.g., liver or kidney dysfunction, hyponatremia], CNS tumor, stroke, migraine, psychiatric eating disorders, etc.) is required. Symptom severity may be quantified using the recently proposed APS severity scale [197]. However, a broad spectrum of brainstem symptoms other than APS (oculomotor disturbances, facial palsy/numbness, ataxia, etc.) has also been described in patients with NMOSD, and all patients should be thoroughly examined for possible brainstem involvement [87, 117]. It should also be taken into account that NMOSD rarely causes diencephalic lesions that may cause hypersomnolence/narcolepsy or a syndrome of inadequate antidiuretic hormone secretion (SIADH). Patients should also be screened for signs of depression, fatigue, and cognitive impairment, and, if these are found, neuropsychological/neuropsychiatric consultation should be considered [9, 23, 60, 149]. Cerebral manifestations may also include headache, and, very rarely, epileptic seizures (with the latter being more frequent in MOG-EM/MOGAD than in AQP4-IgG-positive NMOSD) [8, 52, 152]. Extra-opticospinal symptoms are relatively rare at onset both in AQP4-IgG-positive and in AQP4-IgG-negative patients (e.g., 4% in a German cohort [87] and 9% in a Chinese study [127]), but many patients develop one of them at least once over the course of disease.

NMOSD takes a relapsing course in virtually all AQP4-IgG-positive patients but may be monophasic in AQP4-IgG-negative patients [87]. A diagnosis of monophasic NMOSD should be made with caution, however, since the interval between the first and the second attack varies widely, ranging from just 1 month to more than a decade (median 9 months in a large European cohort [87]). Patients positive for AQP4-IgG should be considered to be at risk of relapse indefinitely.

Accrual of disability in NMOSD is driven mostly by acute clinical attacks. Although ON and myelitis attacks tend to be less severe in MS than in NMOSD on average (at least in AQP4-IgG-positive cases), mild attack-related symptoms should not per se be (mis)taken as evidence against a diagnosis of NMOSD. Indeed, attack severity in NMOSD may range from mild (e.g., tingling, subtle paresis, blurred vision, visual deficiency detectable only by means of low-contrast charts) to extremely severe (e.g., blindness, tetraparesis, respiratory insufficiency). Severe visual deficiency (Snellen score < 20/200 for high-contrast visual acuity) during acute attacks is frequent in AQP4-IgG-positive NMOSD (and MOG-EM/MOGAD) but rare in MS. Whereas persistent severe motor impairment after acute myelitis is more suggestive of AQP4-IgG-positive NMOSD than of MOG-EM/MOGAD, persisting sphincter disturbance/sexual dysfunction is more commonly seen in MOG-EM/MOGAD, as the latter disorder more often affects the conus than does NMOSD [36]. While significant chronic progressive deterioration of symptoms independent of or in addition to clinical relapses frequently occurs in MS, such a course of disease is highly atypical and should be considered a red flag in NMOSD [231] (as well as MOG-EM/MOGAD [84]). However, growing evidence from electrophysiological, radiological, optical coherence tomography (OCT), histopathological, and laboratory studies suggests a role for at least subtle relapse-independent CNS (including retinal) damage in NMOSD (best shown for AQP4-IgG-positive patients) [1, 3, 50, 57, 94, 148, 178, 218].

Even after a single attack severe, permanent disability can remain, especially if the attack is not treated immediately and properly (see Part 2 of this article series for details) [112]. To prevent accrual of disability from repeated attacks, long-term immunotherapy is essential (see Part 2 of this article series) [200]. Especially if left untreated, NMOSD may result in considerable disability, reduction in quality of life, and increased risk of death from acute and chronic complications, and may impose a substantial burden on patients and caregivers. NMOSD causes high societal costs [9, 59, 185]. Respiratory insufficiency due to brainstem or high cervical lesions and urosepsis due to bladder dysfunction count among the most common causes of death from NMOSD. However, mortality rates have dropped significantly since the 1980s and 1990s, most likely reflecting earlier diagnosis and improvements in treatment [32, 87, 139, 229].

As contralateral ON may occur in the initially non-affected eye with a delay of some days or a few weeks in NMOSD, repeat ophthalmological examinations should be performed in patients originally presenting with unilateral ON to document the full extent of neurological damage associated with an attack. This is important, since undocumented deficits may later be mistaken for remnants of additional, unrecognized/subclinical attacks and influence treatment decisions. More generally, ON, spinal cord, brainstem, or encephalitis lesions may occur successively during the course of one and the same attack. Repeat neurological and ophthalmological examinations should thus be generally considered and are mandatory in case of new symptoms.

## How should NMOSD be diagnosed?

**Fig. 1:**
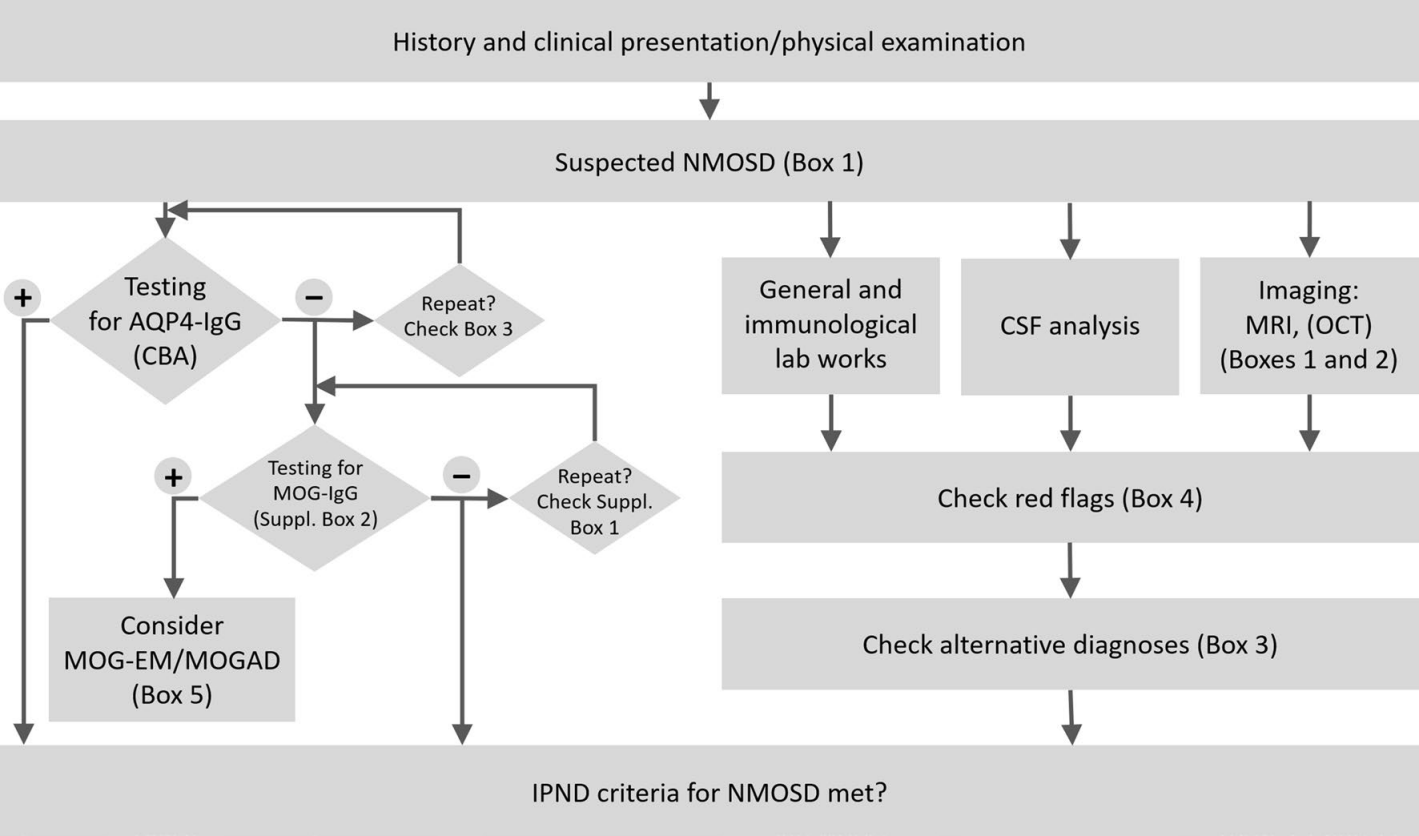
Proposed algorithm for the diagnosis of NMOSD. *AQP4-IgG* aquaporin-4 immunoglobulin G antibodies, *CSF* cerebrospinal fluid, *MOG-EM/MOGAD* myelin oligodendrocyte glycoprotein (MOG) antibody-associated encephalomyelitis/MOG autoantibody-associated disease, *MRI* magnetic resonance imaging, *OCT* optic coherence tomography

The initial workup in patients with suspected NMOSD must include a detailed medical history and an extended physical and neurological examination. Additional mandatory diagnostic tests include cranial and spinal MRI and serum AQP4-IgG antibody testing. Orbital MRI may be required in selected patients (see section on MRI diagnostics for details). Lumbar puncture is highly recommended for differential diagnostic purposes. Optionally, OCT may be performed and evoked potentials assessed. Fig. 1 shows a proposed diagnostic algorithm.

We strongly recommend that NMOSD be diagnosed according to the revised diagnostic criteria proposed by the International Panel for NMO Diagnosis (IPND) in 2015 [228]. These criteria take into account clinical and MRI findings and the AQP4-IgG serostatus and allow NMOSD to be diagnosed after only a single clinical event. Use of the 2006 criteria [230] is strictly discouraged, since it may result in significant underreporting of NMOSD [190]. The 1999 criteria did not yet take into account AQP4-IgG serostatus and must not be used [229].

In patients with a positive AQP4-IgG serostatus, at least one of the six core characteristics listed in Box 1 must be present before a diagnosis of definite NMOSD can be made (note that the definition of core characteristics 5 and 6 includes not only clinical but also MRI findings), and alternative diagnoses that might better explain the patient’s symptoms must be excluded. This is different from previous criteria, which required a history of both acute ON and acute myelitis [230].

**Box 1:** Core characteristics of NMOSD according to Wingerchuk et al. [228]

1. Acute optic neuritis
2. Acute myelitis
3. Acute area postrema syndrome (APS)^#^
4. Acute brainstem syndrome other than APS
5. Symptomatic narcolepsy or acute diencephalic clinical syndrome with NMOSD-typical diencephalic lesion on MRI (see Box 2)
6. Acute cerebral syndrome with NMOSD-typical brain lesion on MRI (see Box 2)

^#^Episode of otherwise unexplained hiccups and/or nausea and vomiting (lasting for at least 48 h or with MRI evidence of a dorsal brainstem lesion)

To make a diagnosis of NMOSD in patients with negative or unknown AQP4-IgG serostatus, at least two of these core characteristics must have occurred at least once, either with a single or, successively, with multiple clinical attacks (i.e., dissemination in space but not dissemination in time is required). In addition, at least one of the core clinical characteristics present must be acute ON, acute myelitis, or APS. Furthermore, the typical MRI criteria (specified in Box 2) must be met. Again, alternative diagnoses that might better explain the patient’s symptoms must be ruled out.

**Box 2:** Typical magnetic resonance imaging findings (T2 unless otherwise noted) in NMOSD (modified from Wingerchuk et al. 2015) [228]

Optic neuritis: Normal cerebral MRI (or only nonspecific white matter lesions), *or* longitudinally extensive optic nerve lesion (≥ half of the length of the optic nerve or involving optic chiasm; T2 or T1/Gd)
Myelitis: Intramedullary lesion ≥ 3 contiguous VS (LETM), *or* focal atrophy ≥ 3 contiguous VS in patients with a history of acute myelitis
Area postrema syndrome: Lesion in the dorsal medulla oblongata/area postrema
Other brainstem syndrome: Periependymal brainstem lesion (4th ventricle)
Diencephalic syndrome : Periependymal lesion (3rd ventricle), *or* hypothalamic/thalamic lesion
Cerebral syndrome: Extensive periependymal lesion (lateral ventricle; often with Gd), *or* long (> 1⁄2 length), diffuse, heterogeneous or edematous corpus callosum lesion, *or* long corticospinal tract lesion (unilateral or bilateral, contiguously involving internal capsule and cerebral peduncle), *or* large, confluent (unilateral or bilateral) subcortical or deep white matter lesion

*Gd* gadolinium, *LETM* longitudinally extensive transverse myelitis, *MRI* magnetic resonance imaging, *VS* vertebral segments

According to the IPND criteria, a diagnosis of NMOSD can be made also in patients with unknown AQP4-IgG serostatus using the same set of criteria as for patients with negative AQP4-IgG serostatus. However, we recommend that such patients should be tested for AQP4-IgG at the earliest possible time, as long-term treatment options may depend on a patient’s serostatus (see Part 2 for details).

In accordance with a proposal made by the IPND [228], we recommend that a diagnosis of relapsing disease should be made in the event that a new attack occurs more than 4 weeks after onset of the initial attack. Additional symptoms that newly emerge within 4 weeks after onset of an attack should be considered part of one and the same attack.

## What alternative diagnoses have to be considered?

A number of differential diagnoses (Box 3) and red flags (Box 4), i.e., clinical, laboratory, or imaging findings that should prompt thorough investigation for competing diagnoses, have to be considered before a definite diagnosis of NMOSD can be made.

**Box 3:** Selected alternative/differential diagnoses for NMOSD

*Non-infectious inflammatory diseases* Relapsing and progressive MS, MOG-EM/MOGAD, neurosarcoidosis, Behcet's disease, rheumatic diseases (e.g., Sjögren’s syndrome*, systemic lupus erythematosus*, overlap syndromes, systemic vasculitis, primary CNS vasculitis), anti-NMDA receptor encephalitis, anti-GFAP-associated encephalomyelitis, paraneoplastic neurological syndromes** (e.g., anti-CV2/CRMP5 [88], anti-GAD65, anti-Hu, anti-Ri, anti-amphiphysin, and, in patients with a diencephalic syndrome, anti-Ma2/Ta-IgG, anti-IgLON5 encephalitis), para-/postinfectious myelitis, para-/postvaccinal myelitis, IgG4-related disease, Susac syndrome, CLIPPERS
*Infectious diseases* Viral myelitis (e.g., varicella zoster virus, enteroviruses, herpes simplex virus, cytomegalovirus, Epstein-Barr virus, West Nile virus, HIV, HTLV 1/2, tick-borne encephalitis virus, poliovirus), neurotuberculosis, neurosyphilis, neuro-borreliosis, *Bartonella henselae*, other rare infections [225]
*Vascular diseases* Spinal cord infarction, spinal dural arteriovenous fistula, anterior/posterior ischemic (including non-arteritic) optic neuropathy, sinus thrombosis (bilateral papilledema), CADASIL
Neoplastic diseases*** CNS lymphoma, intramedullary tumors (e.g., ependymoma and astrocytoma, hemangioblastoma, rarely others [182])
*Genetic and metabolic diseases* E.g., vitamin B12 [81], folate, vitamin E, copper or biotinidase deficiencies [133], Leber's hereditary optic neuropathy [58, 60, 115, 133, 213], leukodystrophies (including Alexander disease)
*Other diseases* Idiopathic intracranial hypertension (bilateral papilledema); traumatic spinal cord, brain, brainstem or optic nerve damage, compressive myelopathy

*CADASIL* cerebral autosomal dominant arteriopathy with subcortical infarcts and leukoencephalopathy, *CLIPPERS* chronic lymphocytic inflammation with pontine perivascular enhancement, *CSF* cerebrospinal fluid, *GAD* glutamic acid decarboxylase, *GFAP* glial fibrillary acidic protein, *HIV* human immunodeficiency virus, *HTLV* human T-lymphotropic virus, *MS* multiple sclerosis, *NMDA* N-methyl-d-aspartate

*Cave: May coexist with AQP4-IgG-positive NMOSD. **Presence of AQP4-IgG does not per se exclude a paraneoplastic nature of NMOSD; however, paraneoplastic cases are rare. ***Extensive diagnostic workup obligatory prior to spinal cord biopsy

**Box 4:** Red flags to be considered before making a diagnosis of NMOSD (modified from Wingerchuk et al. 2015 [228])

*Clinical and laboratory features* Progressive overall clinical course (neurologic deterioration unrelated to attacks; consider MS) Time to attack nadir < 4 h (consider ischemia/infarction) or continual worsening for > 4 weeks from attack onset (consider sarcoidosis or neoplasm) Partial TM, especially when not associated with LETM MRI lesion (consider MS) AQP4-IgG positivity only in the CSF, not in the serum (can be true positive in very rare cases; e.g., if serum testing is hampered by strong background staining, or shortly after PLEX; always consider retesting of serum and CSF in an alternative assay) AQP4-IgM and/or AQP4-IgA positive, but AQP4-IgG negative (clinical significance unknown; not sufficient for making a diagnosis of seropositive NMOSD) “Double positivity” for AQP4-IgG and MOG-IgG (extremely rare/implausible; repetition of both tests recommended in all cases) Presence of CSF-restricted OCB (present in ≤ 20% of cases of NMO vs > 90% of MS [lower in Asian and other populations]) Presence of a bi- or trispecific MRZ reaction (present in around 67% of MS patients, virtually absent in NMOSD)
*Neuroimaging findings* Brain imaging features (T2-weighted MRI) suggestive of MS (MS-typical): Lesions with orientation perpendicular to a lateral ventricular surface (Dawson fingers), *or* lesions adjacent to lateral ventricle in the inferior temporal lobe, *or* juxtacortical lesions involving subcortical U-fibers, *or* cortical lesions Spinal cord characteristics more suggestive of MS than NMOSD: Lesions < 3 complete vertebral segments (sagittal T2), *or* lesions located predominantly (> 70%) in the peripheral cord (axial T2), *or* diffuse, indistinct signal change (T2, seen with longstanding or progressive MS) Lesions with persistent (> 3 months) Gd enhancement (suggestive neither of NMOSD nor MS) or persistent Gd enhancement despite immunotherapy (consider tumor/lymphoma or vascular malformation) Brain linear perivascular radial Gd enhancement (consider GFAP-IgG-associated astrocytopathy and, possibly, neurosarcoidosis, vasculitis, lymphoma)
*Comorbidities* Sarcoidosis, established or suggestive findings thereof (e.g., mediastinal lymphadenopathy, fever and night sweats, elevated serum ACE or soluble IL2R levels [153, 163], leptomeningeal enhancement) Cancer, established or with suggestive clinical, radiologic, or laboratory findings thereof; consider also lymphoma or paraneoplastic disease (e.g., CV2/CRMP5-associated optic neuropathy [88] and myelopathy, or anti-Ma-associated diencephalic syndrome) Chronic infection, established or with suggestive findings thereof (e.g., HIV, syphilis, Tb)

*ACE* angiotensin-converting enzyme, *CSF* cerebrospinal fluid, *IL2R* interleukin-2 receptor, *LETM* longitudinally extensive transverse myelitis, *MRZ* measles/rubella/zoster virus reaction, i.e., intrathecally produced antibodies (positive antibody index) against at least two of these three viral agents, *MS* multiple sclerosis, *OCB* oligoclonal bands, *PLEX* plasma exchange, *Tb* tuberculosis, *TM* transverse myelitis

The most important antibody-related differential diagnosis of NMOSD is MOG-EM/ MOGAD [84, 91, 132, 134]. A first set of diagnostic criteria for MOG-EM/MOGAD has recently been proposed by an international panel of experts [77] (Box 5), together with a list of red flags (Supplementary Box 1), which we recommend be checked in all patients with signs and symptoms suggestive of that disease. Of note, some patients may meet both the diagnostic criteria for NMOSD and those for MOG-EM/MOGAD, due to the substantial clinical and radiological overlap between the two diseases. However, NEMOS strongly recommends that AQP4-IgG-negative patients who meet the criteria for MOG-EM/MOGAD [77, 78] should no longer be assigned the diagnosis of NMOSD, as convincing evidence now suggests that MOG-EM/MOGAD is a pathogenetically, clinically, and prognostically distinct disorder in its own right, possibly with different treatment needs [75, 77, 78, 84, 86, 154]. The recommended indications for MOG-IgG testing can be found in Supplementary Box 2.

**Box 5:** Diagnostic criteria for MOG-EM/MOGAD according to [77, 78] (to be evaluated in all patients meeting the IPND criteria for NMOSD without AQP4-IgG)

All criteria must be met:
1. Monophasic or recurrent acute ON, myelitis, brainstem encephalitis or encephalitis, or any combination of these syndromes
2. MRI or, only in patients with isolated ON, electrophysiological (VEP) findings that are compatible with an inflammatory demyelinating CNS disease
3. Detection of MOG-IgG antibodies in serum by means of a CBA using human full-length MOG as target antigen
In addition, red flags must be checked (see Supplementary Box 1); if red flags are present, the positive MOG-IgG laboratory result should be confirmed in an alternative, methodologically non-identical CBA (or, only if such an assay is not available, at least in a second serum sample)

*ON* optic neuritis, *VEP* visual evoked potentials, *CBA* cell-based assay

As coexistence of AQP4-IgG-positive NMOSD and MOG-EM/MOGAD is considered to be extremely rare [86, 121] and false-positive serological results still occur, patients meeting the diagnostic criteria for both of these two conditions should be reviewed at a specialized center and their "double-positive" antibody status reconfirmed.

The most important differential diagnosis in AQP4-IgG- and MOG-IgG-negative (“double-negative”) patients with NMOSD is MS. However, several other rare autoimmune, paraneoplastic, neoplastic, infectious, metabolic, and vascular diseases that can mimic NMOSD have to be taken into consideration as well (Box 3). In all patients with double-negative NMOSD, neurosarcoidosis should be considered, especially (but not exclusively) in those presenting with longitudinally extensive transverse myelitis (LETM) [40]. Myelitis in NMOSD may also mimic spinal cancer or lymphoma and vice versa; whenever possible, AQP4-IgG (and MOG-IgG) should be tested and NMOSD excluded before a decision is made to perform spinal cord biopsy—a procedure that can leave patients severely disabled—for suspected spinal neoplasia [180]. For a comprehensive overview of relevant differential diagnoses, see also [105, 110, 116, 214].

Of note, coexistence of NMOSD with any of these differential diagnoses may rarely occur and has to be taken into consideration, both in AQP4-IgG-positive and AQP4-IgG-negative patients. Most importantly, coexisting connective tissue disorders (CTD) and other rheumatic disorders, such as systemic lupus erythematosus, antiphospholipid syndrome, Sjögren’s syndrome, or Sharp syndrome, are not uncommon in patients with (usually AQP4-IgG-positive [87]) NMOSD, suggesting a shared autoimmune predisposition, and must per se not be taken as sufficient reason to reject the diagnosis of NMOSD [7, 65, 74, 87, 167, 208]. AQP4-IgG-positive NMOSD has also been reported in association with other autoimmune disorders such as myasthenia gravis (MG) [80, 97, 123, 138], celiac disease [13, 73], and autoimmune thyroid disease. An interdisciplinary approach is recommended in such cases. Co-existing MG should be considered not only in patients with limb paresis that becoms progressively worse during periods of physical activity but also in patients with ocular weakness, respiratory insufficiency, dysphagia, dysarthria, or dysphonia. Overall, signs of co-existing autoimmunity have been reported in 25–40% of patients with AQP4-IgG-positive NMOSD in some cohorts [74, 167, 208].

Although a number of patients with AQP4-IgG-positive NMOSD and co-existing cancer (with AQP4 expression within the tumor tissue in some) have been reported [166], the disorder is not tumor-associated in the vast majority of cases [87, 192]. While tumor screening may in fact be indicated in individual patients, depending on the presence of risk factors or symptoms suggestive of an underlying or co-existing tumor, general screening of all patients with newly diagnosed AQP4-IgG-positive NMOSD for cancer—especially by means of invasive or otherwise potentially harmful methods—is currently not recommended. Male sex, age > 45 years, and presentation with nausea and vomiting have been identified as risk factors for tumor association in European AQP4-IgG-positive patients [192].

All patients with suspected NMOSD attacks should be examined for signs and symptoms of infection. However, it should be taken into account that the onset of NMOSD (and MOG-EM/MOGAD [85]) is not uncommonly preceded by  a common cold or other infection [87]. This can lead to false suspicion of CNS infection. Very rarely, NMOSD (as well as MOG-EM/MOGAD [67, 85]) first occurs following vaccinations [4, 53]. It is unclear whether infection or vaccination induces a de-novo autoimmune reaction in these patients (e.g., by molecular mimicry) or rather acts as a non-specific inflammatory trigger causing clinical exacerbation in patients with pre-existing but previously clinically latent NMOSD. Infections also frequently precede relapses in both NMOSD and MOG-EM/MOGAD; taking into account both the first attack and subsequent attacks, NMOSD attacks were preceded by infections in around 30% of AQP4-IgG-positive and 20% of AQP4-IgG-negative cases in a large European study [87].

## Practical recommendations—antibody testing

AQP4-IgG testing is recommended in all patients with clinical or clinico-radiological findings (either present or previous) that would permit the diagnosis of “NMOSD with positive AQP4-IgG serostatus” in the case of a positive test result according to the IPND criteria, i.e., in all patients with one of the core characteristics listed in Box 1, and in all patients diagnosed with “NMOSD with unknown AQP4-IgG serostatus” according to the IPND criteria. In all other cases, the decision on whether or not to test for AQP4-IgG testing should be made individually; however, broad, unselected screening of patients with MS that do not meet the above-mentioned criteria is discouraged, especially in regions of the world in which NMOSD accounts only for a small proportion of IDD cases, since this practice may result in an unfavorable ratio of false-positive to true-positive results. Typical indications for MOG-IgG testing are listed in Supplementary Box 2. Both AQP4-IgG and MOG-IgG (to rule out MOG-EM/MOGAD in patients meeting the IPND criteria for “NMOSD with AQP4-IgG-negative serostatus”) must be tested by means of cell-based assays (CBA). The assay must employ either fixed or live cells expressing conformationally intact, full-length recombinant human AQP4 or MOG protein, respectively, as antigenic substrate and mock-transfected (or, at minimum, non-transfected) cells as control substrate [77, 78, 82, 90, 175, 228].

Formalin-fixed CBA are easier to standardize, are commercially available and thus widely accessible, and can be utilized at all routine laboratories familiar with basic immunofluorescence techniques; in contrast, the technically more demanding live CBA are offered only by a few specialized laboratories. However, live CBA have been found to be slightly more sensitive in some studies, especially for detecting MOG-IgG [44, 175, 223, 224]. Both types of assays may be used.

Immunohistochemistry (IHC) using rodent or primate CNS tissue sections as substrate, enzyme-linked immunosorbent assays (ELISA), fluorescence-based immunoprecipitation assays (FIPA), Western blot assays, and radioimmunoprecipitation assays (RIA) have all been used in the past but are thought to be less sensitive and/or specific than CBA and should therefore no longer be employed for routine clinical testing [71, 77, 78, 90, 160, 228]. IHC-based assays for detecting AQP4-IgG [70, 90, 125] may be used exceptionally if testing by CBA is strictly not available, neither locally nor by shipment to specialized centers, as is still the case in some regions of the world; in that event, confirmatory testing by means of a CBA should be striven for and should be performed as soon as it becomes available. The use of IHC assays for detecting MOG-IgG is strongly discouraged. Assays using peptides instead of full-length protein as antigens lack specificity and thus are not suitable to make a diagnosis of NMOSD or MOG-EM/MOGAD and must no longer be used [71, 77, 78, 90, 228].

The timing of antibody testing is important, since procedures given for attack treatment (glucocorticosteroids, plasma exchange [PLEX], immunoadsorption [IA]), immunosuppressants, intravenous immunoglobulins) or attack prevention may rarely [48, 66] cause false-negative or ambiguous results. Therefore, samples for antibody testing should ideally be taken before treatment is commenced. As AQP4-IgG and MOG-IgG are produced mainly extrathecally, serum should be used for AQP4-IgG determination as biomaterial of choice [72, 90, 228].

Testing of CSF for AQP4-IgG or MOG-IgG may theoretically be helpful in the very rare cases in which non-specific, coexisting serum antibodies cause a strong background signal that may potentially mask a weak AQP4-IgG- or MOG-IgG-specific signal or if testing is performed shortly after PLEX or IA. In fact, a few reports exist on patients positive for AQP4-IgG or MOG-IgG only in the CSF but not in serum [122, 131]. In most of these cases, however, the results were not independently confirmed and/or no sufficiently large control cohorts were included, so the data remain controversial. Importantly, the current diagnostic criteria recommend the use of serum assays [77, 228]. If, deviating from this recommendation, in rare selected cases a provisional diagnosis is made after consultation with a specialized center based on CSF-only positivity, retesting of the CSF and serum sample in a methodologically different assay as well as confirmatory testing of follow-up serum samples is highly advisable.

The specimen analyzed (serum, CSF), the assay type (e.g., live CBA, fixed CBA, ELISA, tissue-based assay) and the assay manufacturer or performing laboratory, as well as the test result (positive, negative, grey area) and, if available, the titer and cut-off, should be documented in the discharge letter (e.g., “Serum AQP4-IgG positive (12-Sept-2022; titer 1:320, cut-off ≥ 1:10; fixed cell-based assay [Euroimmun])”. In addition, the disease status (acute attack, remission) and treatment status at the time of testing should be documented, especially in the event of a negative test result, since samples obtained during remission or under treatment may occasionally be falsely negative or unequivocal and warrant retesting, as mentioned above [66, 223, 234].

The current diagnostic criteria for seropositive NMOSD strictly require the presence of AQP4-specific antibodies of the IgG isotype [77, 90, 228]. Seropositivity only for IgM and/or IgA antibodies to AQP4 must not be considered sufficient, as the pathogenetic and clinical relevance of such findings is unknown. Among other factors, assay specificity depends on the method used to detect patient IgG bound to the test substrate. As detecting patient IgG by use of so-called H + L-chain-specific secondary antibodies carries the risk of occasional false-positive results due to cross-binding of this type of antibodies to IgM and IgA, so-called Fcγ-specific (or, alternatively, IgG1-specific) secondary antibodies should preferentially be used[77, 90]. Information about the exact method used in a particular assay can be obtained from the manufacturer or the performing laboratory. A summary of methodological recommendations can be found in Supplementary Box 3.

As AQP4-IgG and MOG-IgG titers may vary significantly over time, depending on disease activity and treatment [66, 90, 108], and because of the potential differences in sensitivity between assays, retesting (preferentially by means of a standardized live CBA, if available) should be considered in seronegative patients if NMOSD or MOG-EM/MOGAD is still suspected, ideally during a treatment-free interval or during an acute attack. Given the fact that no serological assay is 100% specific and taking into account the important and often life-long diagnostic and therapeutic consequences of a positive test result, positive results should ideally be confirmed, either in a second, methodologically different cell-based assay or, if such an assay is not available, at least in a second sample, especially in the case of low-titer results (e.g., at cut-off or only one dilution step above cut-off) or if a non-approved (in-house) assay was used. Confirming a positive test result is highly recommended if one or more red flags (Box 4 and Supplementary Box 1) are present [77, 219, 228]. As the currently available serological tests for MOG-IgG (especially in patients with low titers) are of lower specificity than those for AQP4-IgG, this is particularly important before a diagnosis of MOG-EM/MOGAD is made and seronegative NMOSD or MS consequently dismissed [77].

## Practical recommendation—CSF analysis

Analysis of cerebrospinal fluid (CSF) is not formally required to make a diagnosis of NMOSD according to the current diagnostic criteria; however, we strongly recommend lumbar puncture in patients with suspected NMOSD, since CSF analysis can support a diagnosis of NMOSD and help to distinguish the disorder from MS and other differential diagnoses.

Basic CSF examination should include determining oligoclonal bands, CSF white cell counts (WCC), CSF WCC differentiation (cytology), CSF erythrocyte counts, CSF total protein levels, CSF L-lactate levels, and CSF and serum glucose levels. However, we recommend to assess also IgG, IgM, IgA and albumin CSF/serum ratios (in combination with Reiber’s diagrams [173, 174, 226]), IgG/IgM/IgA fractions, and, if available, the so-called measles, rubella, and zoster virus (MRZ) reaction [68, 69, 76, 83]. The use of Link’s IgG index is discouraged due to limited specificity, especially in patients with moderate to severe blood‒CSF barrier dysfunction). While CSF-specific oligoclonal IgG bands are a diagnostic mainstay of MS (present in > 95% of continental European patients, lower frequency in Asian patients) and persist over time, irrespective of clinical disease activity, they are absent in the majority of patients with AQP4-IgG-positive NMOSD (present in around 25% at first LP in [87] and in only ~20% of acute samples and 10% of remission samples in [79]), AQP4-IgG-negative NMOSD (present in around 35% at first LP in [87]), or MOG-EM/MOGAD (~ 10%) and may be present only transiently [76, 79, 83, 87]. CSF WCC ≥ 50/µl, CSF neutrophils (occasionally also eosinophils), and an albumin CSF/serum ratio (QAlb) > 12 × 10^–3^ are all atypical for MS but not uncommon in AQP4-IgG-positive NMOSD (WCC ≥ 50/µl in ~ 15%; neutrophils present in ~ 50%; and QAlb > 12 × 10^–3^ in ~ 30% of all patients with acute attacks and in as many as 50% of those with blood–CSF barrier dysfunction) and MOG-EM/MOGAD (WCC ≥ 50/µl in 46% with acute myelitis, but only very rarely during acute ON; neutrophils in ~ 60% during acute myelitis and ~ 25% during acute ON; QAlb > 12 × 10^–3^ in 24% of all patients and 43% of those with elevated QAlb during acute myelitis, but only rarely during acute ON) [76, 79, 83]. A positive MRZ reaction (as defined by a positive antibody index against at least two of these three viral antigens), which is present in ~ 67% of patients with MS and is considered the most specific laboratory marker of MS known so far, was absent in virtually all patients with AQP4-IgG-positive or -negative NMOSD tested so far (but also in those with MOG-EM/MOGAD) [68, 69, 76, 83]. In contrast to MS, CSF L-lactate levels were mildly increased in an AQP4-IgG-positive NMOSD cohort (in ~ 45% during acute myelitis and ~ 20% during acute ON; 0% during remission; median 2.9 mmol/l during acute attacks, range 2.1–6.8, in a European cohort[79]), similar to what is seen in MOG-EM/MOGAD (~ 25% in patients with acute myelitis, median 2.5 mmol/l, range 1.9–4.43, > 3 mmol/l in around 10%; rarely in acute ON) [76, 79, 83]. It is important to note (a) that the frequency and extent of CSF abnormalities, as detected by lumbar puncture, depends strongly on the lesion site (spinal cord > brain > optic nerve), reflecting both the rostrocaudal CSF gradient and lesion volume, and (b) that CSF findings may be normal, even during attacks, particularly often in patients with acute ON [76, 79, 83]. AQP4-IgG (as well as MOG-IgG) is often detectable also in the CSF, but antibody indices are usually negative, indicating an extrathecal origin [72, 86]. As mentioned above, the current diagnostic criteria for NMOSD (as well as those for MOG-EM/MOGAD) require serum AQP4-IgG positivity; CSF AQP4-IgG positivity alone is currently not considered sufficient.

## Practical recommendation—other laboratory tests

Further laboratory tests can help to identify alternative diagnoses and/or concomitant autoimmune diseases. Besides basic examinations of blood (differential blood count, serum chemistry, and coagulation) and urine, this should include serological examinations for CTD (e.g., ANA, ENA, anti-ds-DNA-antibodies, c/pANCA, cardiolipin antibodies). Further assessments should be considered to exclude hypovitaminosis, other metabolic disorders, infections, autoimmune encephalitis, and neoplastic and paraneoplastic disorders (e.g., anti-collapsin response mediator protein-5 [CRMP5/CV2] antibody-associated optic neuropathy and myelopathy or anti-Ma-associated diencephalic syndrome) (see Box 3 for details).

## Practical recommendations on imaging—MRI

MRI examinations are an essential part of the clinical workup for diagnosis and monitoring of NMOSD [199]. The initial MRI serves as an important baseline examination and shall cover both the brain and the spinal cord. MRI is also essential for distinguishing NMOSD from other CNS conditions.

If available, (fat-suppressed) MRI of the orbits is highly recommended, since it allows more precise depiction of the optic nerve than routine brain MR imaging. It may be required in patients with negative or unknown serostatus if making a diagnosis of NMOSD depends on the presence of longitudinally extensive optic neuritis (LEON) or an optic nerve lesion involving the optic chiasm (see Box 2) and conventional brain MRI does not allow reliable assessment of lesion extension. Particular notice should be paid to lesion location and extension, the presence or absence of optic head swelling, perineural gadolinium (Gd) enhancement, chiasmal lesions, and lesions of the optic tracts. While NMOSD more often affects the posterior (intracranial) part of the optic nerve, lesions in MS and MOG-EM/MOGAD are more frequently located in the anterior portion [196]. Swelling of the optic nerve disk/head on orbital MRI is typically seen in MOG-EM/MOGAD, may infrequently occur in AQP4-IgG-positive NMOSD, and is very rare in MS [171], which typically manifests with retrobulbar ON. LEON is frequent in both NMOSD and MOG-EM/MOGAD but typically absent in MS [140, 171]. The optic chiasm and, more rarely, the optic tracts (often bilaterally [171]) may be affected as well in AQP4-IgG-positive NMOSD, whereas these structures are mostly spared in MS [171]; according to recent data, chiasmal lesions are also not uncommon in MOG-EM/MOGAD and then often extend from the anterior part of the optic nerve to the chiasm [84, 203]. Simultaneous bilateral optic nerve involvement is rather infrequent at onset in AQP4-IgG-positive NMOSD (~ 15% in a European cohort [87]) but may occur later in the disease course. It is more common in seronegative NMOSD and in MOG-EM/MOGAD [84, 87] and relatively rare in MS. Gd-enhanced imaging of the optic nerve is recommended for differential diagnostic purposes. Enhancement of the perineural optic nerve sheath strongly favors a diagnosis of MOG-EM/MOGAD over NMOSD or MS. Demonstration of optic nerve Gd enhancement may also facilitate the discrimination between true relapses and pseudorelapses (defined as worsening or re-occurrence of pre-existing neurological symptoms caused by systemic metabolic changes rather than immune-mediated mechanisms) by providing supportive information [199]. Of note, recent studies found Gd-enhancing lesions of the optic nerve in 20–50% of inter-attack MRI scans, i.e., in asymptomatic patients [159, 193]. In one study, these lesions were mainly located to sites of previous ON attacks, whereas new asymptomatic lesions as detected by Gd-enhanced imaging at sites not previously affected were very rare [193]. Asymptomatic lesions were significantly shorter in length (in mm) than attack-related lesions and were not associated with an increased risk for site-specific attacks in the following year in another study [159].

Spinal MRI should, whenever possible, cover the entire spinal cord, including the conus, since lesion site and longitudinal lesion extension can be diagnostically informative. While myelitis is typically longitudinally extensive—extending over three or more complete vertebral segments—in both AQP4-IgG-positive and AQP4-IgG-negative NMOSD (and MOG-EM/MOGAD), as mentioned above, long lesions are extremely rare in MS and, if present, usually result from the coalescence of adjacent lesions. A finding of exclusively new short lesions during an acute attack of myelitis is more common in MOG-EM/MOGAD (being found at least once in up to 50% of cases over the course of disease in a European cohort [84]) than in NMOSD (10–15% [41, 87]). Short lesions may be present also in combination with LETM lesions [87]. When assessing lesion length, it must be taken into account that lesion extension depends to some extent on timing, with short lesions sometimes representing still evolving or already resolving (if MRI is performed late) LETM. If only short spinal cord lesions are detected but the MRI was performed early during an attack and NMOSD is still suspected, a second MRI during the same attack may be considered; this may be particularly helpful in those patients with suspected NMOSD without AQP4-IgG or NMOSD with unknown AQP4-IgG serostatus according to the IPND criteria in whom the diagnosis depends on the presence of a longitudinally extensive spinal cord lesion [228]. Inflammation of the entire spinal cord has mostly been observed in AQP4-IgG-positive NMOSD cases [87], occasionally occurs in MOG-EM/MOGAD [84], and is not seen in MS. Complete resolution of T2 lesions (and especially of spinal lesions) was shown to be significantly less common and the reduction in T2 lesion area on axial spinal cord imaging to be significantly smaller in AQP4-IgG-positive NMOSD (and MS) than in MOGAD [187]. After severe and/or recurrent myelitis attacks, MRI may also show (longitudinally extensive) spinal cord atrophy in NMOSD (but also in MOG-EM and in long-standing MS). The mean upper cervical cord area may be reduced (AQP4-IgG-positive NMOSD > MOG-EM/MOGAD) [25]. Involvement of the lumbar spinal cord and the conus is less common in AQP4-IgG-positive NMOSD and MS, so its presence favors MOG-EM/MOGAD [36].

Spinal MRI should, whenever possible, include both sagittal and axial imaging. AQP4-IgG-positive NMOSD (as well as MOG-EM/MOGAD) predominantly affects the central portion of the spinal cord (with a preserved peripheral dark rim), whereas MS lesions are mostly located in the peripheral portion [99, 161], often involving the lateral and dorsal columns. Sometimes, only the gray matter is affected, resulting in H-shaped T2 hyperintensity on axial images, termed the “H-sign” [33]; however, this occurs more frequently in MOG-EM/MOGAD and is virtually absent in MS. In contrast, lesions in MS often appear cigar-shaped/cylindrical on sagittal images and wedge-shaped on axial images and are typically sharply demarcated [38].

Spinal MRI should also be examined for swelling, gadolinium enhancement, cavitations, and bright spotty lesions (BSL). Spinal cord swelling during acute myelitis attacks is common in NMOSD, but may also be present in MOG-EM [84] and MS. In severe cases of AQP4-IgG-positive NMOSD, T1-hypointense spinal cord lesions and cavitations may occur [99, 161]. Recently, so-called BSL, defined as hyperintense lesions on axial T2-weighted images and sometimes associated with T1 low signal, have been reported to strongly favor a diagnosis of (especially AQP4-IgG-positive) NMOSD in patients presenting with myelitis [27, 161, 169, 181]. Gd enhancement of spinal cord lesions is common in all three diseases, may be ring-shaped in AQP4-IgG-positive NMOSD (as well as in MS) [235], but may also be patchy and irregular [99, 161]. Contrast-enhanced MRI of the spinal cord is not formally required for making a diagnosis of AQP4-IgG-positive NMOSD according to the IPND criteria [228], but we recommend its use for differential diagnostic purposes [145]. Moreover, by increasing the sensitivity of MRI for detecting active lesions, it may be helpful in improving the discrimination of true relapses and pseudorelapses when relapses cannot be adjudicated based on clinical presentation or T2 imaging alone. This said, it should be taken into account as a limitation, however, that Gd enhancement may persist for more than 1 month after an attack in some patients with NMOSD (but Gd enhancement persisting for more than 3 months should be considered a red flag prompting search for alternative causes [227]) and true myelitis attacks with no new Gd-enhancing lesions may occasionally occur[233].

Spinal cord remission MRI scans, if available, should be examined for new subclinical lesions. While clinically silent spinal cord lesions may be present in all three conditions during acute attacks, an accumulation of silent spinal cord lesions on T2-weighted imaging/fluid-attenuated inverse recovery MRI performed during remission (i.e., outside of a relapse and at least 3 months from the last attack), a characteristic feature used in the diagnosis of MS [207], was mostly absent in NMOSD (4%) in a large, mixed adult and pediatric AQP4-IgG-positive cohort in the UK) [21], as well as in MOG-EM/MOGAD (0%) in the same study [21]. New subclinical lesions during remission, although rare, indicated a high risk of imminent relapse in this study [21]. As a caveat, it should be noted that Gd-enhancing spinal cord lesions may be more commonly present during remission. In the N-MOmentum trial, contrast-enhancing lesions occurred independent of clinical disease activity in 15% of patients, probably representing blood–brain barrier damage or subclinical attacks, and were associated with a significantly increased risk of domain-specific attacks in the following year [30, 159]. Moreover, follow-up spinal cord imaging after acute attacks may be justified to assess the efficacy of attack treatment and the presence or absence of spinal cord atrophy.

Spinal nerve root enhancement or myeloradiculitis has been observed in a few patients with AQP4-IgG-positive NMOSD [47, 101, 102, 204, 211], but was recently reported also in MOG-EM/MOGAD [176, 202, 206, 217] and even in individual patients with (pediatric) MS [206]. Importantly, root enhancement should also prompt radiologists to consider spinal cord sarcoidosis as an alternative diagnosis (which may be associated with subpial, leptomeningeal enhancement; LETM; and a “trident sign” on axial imaging).

Brain and/or spinal MRI should always cover the cranio-cervical junction, as upper cervical spinal cord lesions in AQP4-IgG-positive NMOSD (and MOG-EM/MOGAD) frequently extend into the brainstem [5, 161]. MRI should also include contrast-enhanced, thin-section imaging of the brainstem in case signs of symptoms suggestive of brainstem encephalitis are present. Brainstem lesions, which are more common in AQP4-IgG-positive than in AQP4-IgG-negative patients [5, 87], may also occur independently of spinal cord lesions. They often involve the dorsal medulla oblongata (area postrema) in (typically AQP4-IgG-positive) NMOSD and predominantly the pons in MOG-EM/MOGAD. Lesions affecting the middle cerebellar peduncle are more suggestive of MOG-EM/MOGAD (or MS) than of AQP4-IgG-positive NMOSD [136]. Brainstem lesions are often bilateral in AQP4-IgG-positive NMOSD. Diencephalic lesions are suggestive of (especially AQP4-IgG-positive) NMOSD, whereas the deep gray matter (thalami, basal ganglia) is more often affected in MOG-EM/MOGAD.

Importantly, neither a normal brain MRI nor clinically silent lesions should per se be taken as evidence against NMOSD. While a normal brain MRI is not unusual in NMOSD (and, less frequently, may occur also in MOG-EM/MOGAD), especially at onset (both in AQP4-IgG-positive and in AQP4-IgG-negative patients [87]), brain lesions are mandatory to make a diagnosis of MS according to current criteria. Clinically silent brain lesions may accompany acute ON or myelitis attacks (most frequently seen in the corpus callosum, followed by the internal capsule, and the cerebral peduncles in AQP4-IgG-positive patients) [103, 168], but large tumefactive (even bilateral) lesions (as occasionally seen also in MOG-EM/MOGAD and MS), cavitary lesions, and residual T1 lesions (rare in MOG-EM/MOGAD but frequent in MS) may occur as well. As with silent spinal cord lesions, accumulation of silent brain lesions during remission, as frequently seen in MS, is widely absent in AQP4-IgG-positive NMOSD (5%) (and also rare in MOG-EM/MOGAD [4% in a mixed adult and pediatric cohort, 12% in an exclusively pediatric cohort]) [21, 37].

Particular attention must be paid to brain lesion extension and distribution. Like spinal cord and optic nerve lesions, brain lesions tend to be longitudinally extensive in NMOSD (with the best evidence available for AQP4-IgG-positive patients), including corticospinal tract lesions and corpus callosum lesions [45, 99]. An ADEM-like lesion pattern on MRI is suggestive of MOG-EM/MOGAD, not NMOSD, as is a ‘leukodystrophy-like’ MRI pattern (characterized by confluent, largely symmetrical lesions) [51], which mainly occurs in children. MOG-EM/MOGAD is further characterized by so-called fluffy, poorly demarcated/ill-defined T2 lesions (as opposed to well-demarcated lesions in MS) [109]. Periependymal lesions around the lateral, third, and/or fourth ventricle are a common finding in (typically AQP4-IgG-positive) NMOSD and should not be (mis)taken as evidence of MS. The temporal lobe is rarely affected in AQP4-IgG-positive NMOSD [136] but frequently involved in MS (typically the inferior temporal lobe [95, 96, 137]) and in MOG-EM/MOGAD [136].

Contrast-enhanced MRI of the brain is not formally required to make a diagnosis of NMOSD but is recommended, especially at onset, for differential diagnostic purposes. Brain lesions in NMOSD often show patchy, cloud-like Gd enhancement. Gd enhancement may also be seen on T1 imaging alongside extensive periependymal T2 brain lesions [228]. Finally, pencil-thin ependymal Gd enhancement is considered to be relatively specific for AQP4-IgG-positive NMOSD [11]. Leptomeningeal brainstem enhancement and leptomeningeal enhancement accompanying cortical encephalitis on fluid-attenuated inversion recovery (FLAIR) imaging, on the other hand, argue rather against NMOSD (although rare cortical involvement has been reported in patients with positive or unknown AQP4-IgG [107, 201]) and more in favor of MOG-EM/MOGAD [18, 20]. As with spinal MRI, brain MRI examinations conducted very early during an attack may underestimate the peak lesion load, which may not occur until a few days later; follow-up MRI during acute attacks may therefore be advisable, especially in the event of newly emerging symptoms or worsening of attack-related symptoms.

Further MRI findings considered typical for NMOSD according to the current criteria are shown in Box 2. As a caveat, CNS lesions in both NMOSD (~ 15%) and MOG-EM/MOGAD (~ 40% at least once in the course of disease in a European cohort [84]) may formally meet the Barkhof or Swanton criteria for MS in some cases. We strongly recommend consideration of the MRI features listed in Box 4 as ‘red flags’, the presence of which should prompt physicians to critically challenge a diagnosis of NMOSD [99, 228].

Imaging protocols and sequences should be chosen according to the clinical situation (diagnosis vs. monitoring of disease course and/or treatment responses) and with respect to the region of interest (brain, optic nerve/orbits, spinal cord). Generally, 3 T systems should be preferred to those of 1.5 T because of their better resolution. 3D T1 and FLAIR sequences (e.g., 1 mm isotropic) should be preferred over 2D acquisitions. In the case of 2D acquisitions, the slice thickness should not exceed 3 mm. As said before, spinal cord imaging should, if possible, include complete coverage with axial T2 acquisition to increase sensitivity for short lesions [41, 43]. MRI reports should ideally state the total number of lesions and the number of new lesions, together with their dimensions and location in detail (e.g., centralized versus lateral lesions in the spinal cord) [228]. The MRI at onset should include contrast-enhanced T1 images and ideally a diffusion-weighted sequence and a susceptibility-weighted sequence or conventional gradient echo T2* sequence, to separate inflammatory from malignant and vascular processes. Macrocyclic rather than linear Gd agents should be used and unnecessary contrast-enhanced MRI studies avoided to mitigate potential health risks associated with accumulation of Gd in the CNS [49]. Reports on attack-related and chronic-progressive brain atrophy in AQP4-IgG-positive NMOSD exist, but data on the structured involved and the severity vary among studies [50, 63, 135, 209, 221]; currently, monitoring of brain atrophy is not generally recommended outside of clinical studies. Beyond this, the clinical utility of non-conventional MRI (e.g., 7-T MRI, central vein sign, diffusion tensor imaging) for diagnosis, differential diagnosis, and disease monitoring remains to be evaluated [29, 118, 120, 198].

## Practical recommendations—visual acuity, OCT, fundoscopy, and perimetry

Habitually corrected visual acuity (VA) must be assessed in both eyes in all patients presenting with a suspected attack of NMOSD. As subclinical ON damage has been observed in patients with clinical myelitis, brainstem encephalitis, or encephalitis, VA should be assessed irrespective of a patient’s clinical presentation. VA assessment should be performed in a standardized manner by an experienced examiner, ideally by an ophthalmologist or optometrist. Especially if VA appears normal using standard Snellen charts, low-contrast VA, which is exclusively altered in some patients, should be tested in addition, e.g., with the ETDRS (Early Treatment Diabetic Retinopathy Study) contrast chart, SLOAN letters 2.5%. In patients with acute ON, VA should be assessed at presentation, shortly after attack treatment (to inform about the need for potential treatment escalation; see Part 2), and before discharge and should be documented in the discharge letter. VA should be assessed also at all follow-up visits.

OCT is a non-invasive technique to generate high-resolution 2D and 3D images of the neural and vascular components of the retina [46, 151]. OCT is an optional procedure not formally needed to reach a diagnosis of NMOSD. However, as it can provide useful supportive information of differential diagnostic relevance, its use is recommended. If OCT is carried out, a high-resolution macular volume scan should be performed to allow analysis of all macular layers and semi-automatic segmentation of all layers. The peripapillary retinal nerve fiber layer (pRNFL) thickness should be assessed as the most informative parameter by performing a ring scan around the optic nerve. In addition, the combined ganglion cell/inner plexiform layer (GCIPL) thickness, the presence or absence of microcystic macular edema (MME) in the inner nuclear layer (INL), the size and shape of the fovea, and the retinal vessel density (using OCT angiography) may be assessed. All scans must be checked for sufficient quality and for segmentation errors before analysis [184].

In NMOSD, most studies have consistently shown thinning of the RNFL and the GCIPL after ON attacks that is, on average, more severe in AQP4-IgG-positive NMOSD than in MS [12, 150, 172, 186, 216]. Based on retrospective analysis of 11 studies (not all of which distinguished between AQP4-IgG-positive and AQP4-IgG-negative patients), OCT shows average RNFL thinning after ON to 55–83 µm in NMOSD and 74–95 µm in MS, compared to an average RNFL thickness of 93–108 µm in healthy individuals without a history of ON [12]; more profound RNFL atrophy (especially in non-temporal quadrants) than in MS has also been demonstrated in MOGAD in a recent pediatric cohort after the first ON attack [155]. A correlation between RNFL thinning and atrophy of the visual pericalcarine cortex has been found in an almost exclusively AQP4-IgG-positive cohort [221]. OCT may be used as a supportive diagnostic tool already early in the disease course. Notably, 88% of NMOSD eyes displayed RNFL thickness < 60 µm even after just one episode of ON in a mixed AQP4-IgG-positive and -negative cohort [216].

Looking into cross-sectional images in more detail, up to 26% of eyes after ON featured MME in the INL in mixed AQP4-IgG-positive and negative NMOSD cohorts. INL-MME rarely appears after MS-ON (5–6%) and is not observed in healthy controls [12, 186]. Moreover, foveal morphometry revealed a wider and flatter fovea in AQP4-IgG-positive NMOSD than in MS and healthy controls [144].

Fundoscopy may facilitate the differential diagnosis of NMOSD and is thus recommended. Demonstration of papillitis/papilledema and/or retinal hemorrhages renders MOG-EM/MOGAD more likely than NMOSD and MS, in which optic disk edema is both less frequent and, usually, less severe. However, a number of differential diagnoses exist that need to be excluded (see Ref. [14, 82]).

Visual field testing is also recommended, since it can provide supportive differential diagnostic information, help to assess attack severity, and may be important for monitoring the efficacy and extent of treatment-driven or spontaneous attack resolution in patients with acute NMOSD-ON. Perimetric studies have demonstrated non-central scotomas (altitudinal, quadrant, three-quadrant, hemianopsia, and bitemporal hemianopsia) in around 25% of patients with AQP4-IgG-positive NMOSD-ON, whereas MS patients typically exhibit central scotoma [64, 141, 146]. Altitudinal hemianopia is the most common type of non-central scotoma in NMOSD and is rare in MOG-EM/MOGAD but may occur also in double-seronegative ON [64]. Complete visual field loss during acute attacks is more frequent in NMOSD and MOG-EM/MOGAD than in MS.

## Practical recommendations on electrophysiology—evoked potentials

Visual-evoked potentials (VEP) and, to a lesser degree, somatosensory-evoked potentials (SSEP) examinations are helpful in the differential diagnosis of NMOSD and are thus recommended, although neither is formally required to make a diagnosis of NMOSD according to the IPND criteria. As in MS and MOG-EM/MOGAD, ON in NMOSD was associated with prolonged P100 latencies caused by demyelination both in AQP4-IgG-positive and in mixed, AQP4-IgG-positive and AQP4-IgG-negative cohorts [84, 178, 179]. Reduced amplitudes, suggesting axonal damage, or even a complete lack of response are seen more commonly in AQP4-IgG-positive NMOSD and MOG-EM/MOGAD than in MS [84, 178, 179]. VEP evidence of demyelination is considered a sufficient substitute for MRI evidence of CNS demyelination in patients with isolated ON in the current diagnostic criteria for MOG-EM/MOGAD [78].

## What other biomarkers are of interest? New developments

Pathophysiologically, AQP4-IgG-positive NMOSD is characterized by direct damage of astrocytes induced by antibodies against the water channel protein AQP4 and secondary damage to oligodendrocytes and neurons, leading to demyelination and axonal loss [82, 89, 126]. Biomarkers for astrocytic damage and neuroaxonal degeneration may thus potentially be of diagnostic and differential diagnostic impact. Neurofilament light chain (NfL), glial fibrillary acidic protein (GFAP), chitinase 3-like 1 protein (CHI3L1), and glutamine synthetase (GS) have all been found to be increased in the CSF of AQP4-IgG-positive NMOSD patients (and NfL and GFAP also in the serum[1, 61, 98, 183, 222]), especially during acute attacks [1, 31, 111, 205]. Elevated CSF GFAP levels have been occasionally observed also in AQP4-IgG-negative patients [111, 222]). Similarly, elevated CSF levels of the neutrophil-related chemokines CXCL1, CXCL5, and CXCL7, the B-cell chemoattractant CXCL13, the T-cell activation marker soluble CD27 (sCD27) [128, 130, 236], and the proinflammatory cytokine interleukin-6 (IL-6), a key mediator of the immune response in AQP4-IgG-positive NMOSD [26, 42, 215], have been described. Finally, a role for autoantibodies to glucose-regulated protein 78 (GRP78) in the pathogenesis of blood–brain barrier breakdown in both AQP4-IgG-positive NMOSD and MOG-EM/MOGAD has been suggested [194, 195]. However, none of these markers is yet part of the standard diagnostic workup of patients with suspected NMOSD, and we recommend (in the absence of gold standard assays and/or generally accepted cut-offs) that these markers should currently be used mainly in the context of scientific studies.

## Unmet needs and outlook

The 2015 IPND diagnostic criteria for “NMOSD without AQP4-IgG” or “NMOSD with unknown AQP4-IgG serostatus” are based mainly on clinical and MRI features typically found in AQP4-IgG-positive NMO. This is useful in enabling a diagnosis of NMOSD also in patients with a false-negative AQP4-IgG test result and in AQP4-IgG-positive patients in whom AQP4-IgG testing has not yet been performed or who do not have access to AQP4-IgG testing. However, patients with genuinely negative AQP4-IgG serostatus may also meet these criteria, which renders the subgroup heterogenous. Revised criteria should thus stress the need for strictly distinguishing “NMOSD with unknown AQP4-IgG serostatus” and “NMOSD without AQP4-IgG” as different diagnostic categories and should define strategies aiming at identifying patients with potentially false-negative AQP4-IgG results who may be included in the latter category. Moreover, more studies addressing the pathogenesis of seronegative NMOSD and the question of potential pathogenetic, prognostic and/or therapeutic heterogeneity within that category are highly desirable. Demonstration of such heterogeneity might have important implications for nosology and future diagnostic criteria, as it might bring about a need for further diagnostic stratification. Most importantly, future diagnostic criteria for NMOSD should pay particular attention to improving the discrimination of NMOSD and MOG-EM/MOGAD, should explicitly exclude patients with MOG-EM/MOGAD from the category of “NMOSD without AQP4-IgG”, and, accordingly, should make MOG-IgG testing mandatory before a diagnosis of “NMOSD without AQP4-IgG” can be assigned.

Although OCT is of differential diagnostic value in general (and may, according to recent studies [24, 155], help discriminate MOG-EM/MOGAD and MS after a first ON attack), its utility for reliably distinguishing NMOSD from MOG-EM/MOGAD and MS is still limited. Combined scores taking into account structural OCT/OCT angiography parameters (such as, e.g., peripapillary RNFL, combined GCIPL thickness, and retinal vessel density), spatial distribution of OCT alterations, and functional parameters such as high- and low-contrast VA and the application of artificial intelligence and deep learning algorithms may have the potential to increase the discriminatory power of OCT in the future [100, 164]. However, inclusion of OCT in future criteria will be hampered by the limited availability of this technique in many parts of the world. The same is true for the implementation of 7 T MRI, novel advanced MRI sequences, or extended CSF analysis (including MRZ testing). Revised future criteria may yet well exploit these new developments by providing a list of (weigthed) supportive criteria, which could help to increase diagnostic certainty in patients with low-titer AQP4-IgG test results. Combined MRI scores may also help to improve both diagnostic specificity and sensitivity.

## Conclusions

The diagnosis of NMOSD should be made according to the 2015 IPND criteria. The use of up-to-date, standardized CBA is of crucial importance. Both fixed and live CBA have been shown to be highly specific and sensitive for detecting AQP4-IgG; the use of other methods is currently discouraged. Confirmation of positive test results is generally recommended and is mandatory if red flags are present. MRI, CSF, electrophysiological, and OCT analyses can support the diagnosis. For NMOSD with negative or unknown AQP4-IgG status, obligatory MRI criteria exist. Particularly careful attention must be paid to ruling out differential diagnoses, especially in patients with seronegative NMOSD. The most important differential diagnoses include MOG-EM/MOGAD, MS, neurosarcoidosis, paraneoplastic neurological syndromes, and infectious diseases. In part 2 of this article series, we will provide detailed recommendations regarding the treatment of NMOSD.

## Supplementary Information

Below is the link to the electronic supplementary material.

Supplementary file1 (PDF 123 KB)

## Abbreviations

ACE: Angiotensin-converting enzyme
ADEM: Acute disseminated encephalomyelitis
APS: Area postrema syndrome
AQP4: Aquaporin-4
CADASIL: Cerebral autosomal dominant arteriopathy with subcortical infarcts and leukoencephalopathy
CBA: Cell-based assay
CHI3LI: Chitinase 3-like 1 protein
CLIPPERS: Chronic lymphocytic inflammation with pontine perivascular enhancement
CNS: Central nervous system
CSF: Cerebrospinal fluid
CTD: Connective tissue disorder
ELISA: Enzyme-linked immunosorbent assay
ETDRS: Early Treatment Diabetic Retinopathy Study
FIPA: Fluorescence-based immunoprecipitation assay
FLAIR: Fluid-attenuated inversion recovery
GCIPL: Ganglion cell/inner plexiform layer
GAD: Glutamic acid decarboxylase
Gd: Gadolinium
GFAP: Glial fibrillary acidic protein
GRP78: Glucose-regulated protein 78
GS: Glutamine synthetase
HIV: Human immunodeficiency virus
HLA: Human leukocyte antigen
HTLV: Human T-lymphotropic virus
IDD: Inflammatory demyelinating disorders
IgG: Immunoglobulin G
IL-6: Interleukin-6
IL2R: Interleukin-2 receptor
INL: Inner nuclear layer
IPND: International Panel for NMO Diagnosis
LEON: Longitudinaly extensive optic neuritis
LETM: Longitudinally extensive transverse myelitis
MOG: Myelin oligodendrocyte glycoprotein
MOG-EM: MOG encephalomyelitis
MOGAD: MOG antibody-associated disease
MRI: Magnetic resonance imaging
MRZ: Measles/rubella/zoster virus reaction
MS: Multiple sclerosis
NEMOS: Neuromyelitis optica study group
NfL: Neurofilament light chains
NMDA: *N*-Methyl-d-aspartate
NMO: Neuromyelitis optica
NMOSD: Neuromyelitis optica spectrum disorders
OCB: Oligoclonal bands
OCT: Optical coherence tomography
ON: Optic neuritis
PLEX: Plasma exchange
QAlb: Albumin CSF/serum quotient
RIA: Radioimmunoprecipitation assay
RNFL: Retinal nerve fiber layer
sCD27: Soluble CD27
SIADH: Syndrome of inadequate antidiuretic hormone secretion
Tb: Tuberculosis
TM: Transverse myelitis
VEP: Visual evoked potentials
VS: Vertebral segments
