# Supplementary material for: Update on the diagnosis and treatment of neuromyelits optica spectrum disorders (NMOSD) – revised recommendations of the Neuromyelitis Optica Study Group (NEMOS). Part I: Diagnosis and differential diagnosis
Source: J Neurol. 2023 Apr 6;270(7):3341–68. doi: 10.1007/s00415-023-11634-0 (PMC10267280; doi:10.1007/s00415-023-11634-0)
Supplement: Supplementary file 1 — Supplementary file1 (PDF 123 KB) [file 415_2023_11634_MOESM1_ESM.pdf]

Supplementary Box 1. “Red flags” in patients with suspected MOG-EM/MOGAD (consider retesting for MOG-IgG using an alternative, i.e., methodologically non-identical, cell-based assay to exclude a false-positive test result; if not available or in the case of discrepant results consider presentation in a specialized centre) (slightly modified from [7-9])

1. Course of disease

- Continuous, relapse-independent, chronically progressive deterioration (very rare in MOG-IgG-positive patients [23]), including secondary (especially without relapses) and primary chronic progressive MS
- Sudden onset of symptoms, e.g., <4 h from onset to maximum (consider ischemic cause), or continuous worsening of symptoms over weeks (consider tumour, sarcoidosis, etc.)

2. MRI findings

- Dawson's finger-like lesion or lesion adjacent to a lateral ventricle that is ovoid/round or associated with an inferior temporal lobe lesion
- Marked activity in the cranial MRI between attacks with a silent increase in the lesion load (low evidence)

3. CSF findings:

- Bi- or trispecific MRZ reaction (consider MS)[5]

4. Serology

- MOG-IgG titer at or just slightly above the assay-specific cut-off [a]; especially, but not exclusively, when the clinical picture is atypical
- MOG-IgM and/or MOG-IgA positive, but MOG-IgG negative (clinical significance currently unknown/under investigation)
- MOG-IgG positivity only in the CSF, not in the serum [b]
- “Double positivity” for AQP4-IgG and MOG-IgG (extremely rare; repetition of both tests recommended)

5. Miscellaneous

- Clinical or paraclinical findings that indicate a diagnosis other than MOG-EM, NMOSD or MS (e.g., neurotuberculosis, borreliosis, syphilis, sarcoidosis, Behçet's syndrome, subacute combined degeneration of spinal cord, Leber’s hereditary optic neuropathy, vasculitis, CNS lymphoma, gliomatosis cerebri, paraneoplastic neurological syndromes, posterior reversible encephalopathy syndrome, progressive multifocal leukoencephalopathy, evidence of CNS infection [c], GFAP antibodies)

[a] Exception: patients who were previously positive at levels clearly above the cut-off, in which case low-titer results may reflect true (spontaneous or treatment-induced) decline in antibody levels. [b] A number of recent reports exist on patients with MOG-IgG in the CSF but not in the serum; however, until the clinical significance of these findings is better understood and methodological issues clarified, such finding should be considered a red flag and re-testing of both the CSF and the serum sample in a methodologically different assay is recommended. May also be valid in the rare instances in which coexisting serum autoantibodies hamper serum analysis but not CSF analysis (false-negative serum test). [c] Note that CSF findings in MOG-EM may mimic CNS infection with neutrophil pleocytosis, elevated lactate levels and impaired blood–CSF barrier function. [d] May be true positive in the cases in which MOG-EM and unrelated peripheral neuropathy of other cause coexist, including in the context of other autoimmune disorders (as suggested, for example, by the finding of GM1, Caspr2 or NF155 autoantibodies, respectively, in four patients in [14]).

Supplementary Box 2. Recommended indications for MOG-IgG testing in patients# with acute CNS demyelination of putative autoimmune pathogenesis (modified from [7, 8])

2015 IPND criteria for "NMOSD without AQP4-IgG" met (see Boxes 1 and 2 and ref. [8])

or

All of the following three criteria met:

1. Monophasic or recurrent acute ON, myelitis, brainstem encephalitis, or encephalitis, or any combination of these syndromes

and

2. Radiological or - only in patients with ON - electrophysiological (VEP) findings that are compatible with a demyelinating disease of the CNS

and

3. at least one of the following findings (any finding from any category):

MRI: LETM (≥3 VS); *or* longitudinally-extensive atrophy (≥3 VS) after acute myelitis; *or* conus medullaris lesion (especially at the onset of the disease); *or* LEON (e.g. >1/2 of length of optic nerve; see [7, 8] for an overview of definitions in use); *or* perioptic contrast enhancement in patients with ON; *or* normal supratentorial MRI in patients with isolated ON/myelitis/BSTE; *or* large confluent T2 lesions; *or* MRI findings compatible with ADEM; *or* H sign in myelitis

Fundoscopy: ON with papillary oedema/papillitis (strong evidence); *or* retinal haemorrhages (weak evidence)

Laboratory: Neutrophil CSF pleocytosis; *or* CSF white cell count >50/µl; *or* negative OCB at first or repeat LP (only applies to continental European patients); *or* diagnostic criteria for MS met but negative EBV serostatus

Histopathology: Primary demyelination with intralesional complement detection; *or* perivascular demyelination; previous diagnosis of "pattern II MS"

Clinical findings: Simultaneous bilateral acute ON; *or* disease mainly characterized by recurrent ON; *or* unusually high ON frequency; *or* ON with particularly severe acute visual deficit/blindness in one/both eyes; *or* particularly severe or frequent episodes of acute myelitis or BSTE; *or* persistent bladder/rectal/erectile dysfunction after myelitis; *or* area postrema syndrome (etiologically unclear persistent nausea/recurrent vomiting or hiccups >48 hours or with dorsal medullary lesion); *or* diagnosis of "ADEM"; *or* acute respiratory failure; *or* impaired consciousness; *or* behavioural changes; *or* epileptic seizures (radiological evidence of demyelination required; particularly, but not exclusively, when associated with cortical [FLAIR] lesions); *or* onset of illness 4d to approx. 4 weeks after vaccination; *or* history of teratoma [15] or NMDAR encephalitis

Treatment response: Early relapses/flaring-up of attack symptoms after IVMP *or* steroid-dependent symptoms (incl. CRION); *or* unexpected increase in relapse rate after treatment with IFNβ or other MS therapeutics (except ocrelizumab and AZA)

N.b.: The following recommendations are primarily intended for use in adults and adolescents. Indications for MOG-IgG testing in young children need not to be as rigorous as in adults, since MOG-EM is thought to be significantly more frequent among young children with acquired demyelinating disease (up to 70%; frequency declining with age) than in their adult counterparts (≤ 1% in Western countries; probably ≤ 5% in Japan and other Asian countries because of lower MS prevalence), which reduces the risks attached to antibody screening outlined in [7, 8]

Supplementary Box 3. Recommendations regarding the methodology of and specimen selection for AQP4-IgG and MOG-IgG testing and on data interpretation and reporting (modified from [7] and [12])

Assay types

Cell-based assays (IFT/FACS): Recommended (current gold standard); must employ full-length human MOG or AQP4 as target antigen; non-transfected cells or, preferentially, mock-transfected cells are required as control substrate; use of Fc-specific, or preferentially so-called Fc-gamma-specific' (or IgG1-specific) secondary antibodies highly recommended to avoid cross-reactivity with (specifically or non-specifically co-binding) IgM and IgA antibodies.

Immunohistochemistry: Currently not recommended (less sensitive than cell-based assays, limited data available on specificity, sensitivity depends on tissue donor species); if used to detect AQP4-IgG (e.g. as confirmatory test), Fc-specific secondary antibodies adsorbed against tissue donor IgG required in order to avoid cross-reactivity with IgM and IgA or with tissue-bound donor IgG

Peptide-based ELISA, Western blot: currently not recommended for detecting AQP4-IgG, obsolete for detecting MOG-IgG (insufficiently specific)

Biomaterial

Serum: Recommended (specimen of choice); shipment at 4 °C or on dry ice advisable if samples do not arrive within 1–2 days

Cerebrospinal fluid: Not usually required, since AQP4-IgG are produced mostly extrathecally, resulting in lower CSF than serum titers; potentially helpful in rare, selected cases (e.g., strong background due to coexisting high-titer non-AQP4-specific serum antibodies); several recent reports on patients with MOG-IgG only in the CSF but not in the serum exist; however, the specificity and clinical significance of these reports as well as the nosological relationship of MOG-EM with CSF-restricted antibodies to classical MOG-EM is not completely clear (although future recommendations may advocate CSF testing in patients with suspected MOG-EM, this is currently not standard of care and current criteria require serum positivity for MOG-IgG); shipment at 4 °C or on dry ice generally advisable

Immunoglobulin classes

Testing for AQP4/MOG-IgG: Recommended

Testing for MOG/AQP4-IgM and/or AQP4/MOG-IgA: Currently not recommended; additional AQP4-IgM and -IgA antibodies have been described in some AQP4-IgG-positive patients and, correspondingly, MOG-IgM and -IgA antibodies in some MOG-IgG-positive patients; however, the clinical relevance of isolated AQP4- or MOG-IgM or -IgA results is still unknown/under investigation; testing for antibodies of the IgM class requires removal of total IgG from the sample to avoid both false-negative (due to high-affinity IgG displacing IgM) and false-positive (due to IgM anti-IgG_{Fc} rheumatoid factors) results

Data reporting

Immunoglobulin class detected, assay type, antigenic substrate/biomaterial used (including cell line, AQP4 [M1, M23]/MOG isoform and species), titer/concentration/units, assay-specific cut-offs, and performing laboratory should all be documented both in the testing laboratory's report and in the discharge letter (e.g., "Serum AQP4-IgG 1:320 [cut-off ≥ 1:10[a]; assay: fixed CBA, Heidelberg lab; antigen: full-length human M1-AQP4]")

Data interpretation

As with all laboratory tests, positive test results should always be interpreted in the context of the patient's overall presentation; if "red flags" as defined in Box 4 for AQP4-IgG and in Supplementary Box 3 for MOG-IgG are present, retesting of the positive serum sample (or, if no longer available, at least testing of a follow-up serum sample) is recommended; to reduce the potential risk of reproducing false-positive results due to issues inherent to the method employed, use of a second (and, in the case of discrepant results, third) methodologically different cell-based assay is advisable; if in doubt, seek expert advice from a specialized center.

Timing issues

AQP4-IgG and MOG-IgG serum concentrations depend on disease activity (with higher median concentrations during acute attacks than during remission) and treatment status (with lower concentrations while on immunosuppression) and may transiently vanish after plasma exchange; if AQP4-IgG or MOG-IgG, respectively, is negative but AQP4-IgG-/MOG-IgG-related disease is still suspected, retesting during acute attacks, during treatment-free intervals, or 1–3 months after plasma exchange (or IVIG[b]) is recommended. NB: Some cases of monophasic MOG-IgG-positive EM/ADEM in adult patients have been described in which MOG-IgG disappeared permanently following clinical recovery[1, 3, 6, 10, 11, 13].

[a] Note that the cut-off given here is an example only; actual cut-off values are assay-dependent. [b] Generally, pretreatment with IVIG is liable to cause false-negative and false-positive results in antibody assays[4]. A recent study did not find a major effect of IVIG on the sensitivity and specificity of two currently widely used AQP4-IgG and MOG-IgG assays in a relatively small cohort of patients[2], but systematic data are lacking. Abbreviations: ADEM, acute disseminated encephalomyelitis; AQP4, aquaporin-4; CSF, cerebrospinal fluid; ELISA, enzyme linked immunosorbent assay; EM, encephalomyelitis; IVIG, intravenous immunoglobulins; MG, myasthenia gravis, MOG, myelin oligodendrocyte glycoprotein.

References

1. Di Pauli F, Mader S, Rostasy K, Schanda K, Bajer-Kornek B, Ehling R, Deisenhammer F, Reindl M, Berger T (2011) Temporal dynamics of anti-MOG antibodies in CNS demyelinating diseases. Clin Immunol 138:247-254
2. Gruter T, Ott A, Meyer W, Jarius S, Kinner M, Motte J, Pitarokoili K, Gold R, Komorowski L, Ayzenberg I (2020) Effects of IVIg treatment on autoantibody testing in neurological patients: marked reduction in sensitivity but reliable specificity. J Neurol 267:715-720
3. Hoftberger R, Sepulveda M, Armangue T, Blanco Y, Rostasy K, Cobo Calvo A, Olascoaga J, Ramio-Torrenta L, Reindl M, Benito-Leon J, Casanova B, Arrambide G, Sabater L, Graus F, Dalmau J, Saiz A (2015) Antibodies to MOG and AQP4 in adults with neuromyelitis optica and suspected limited forms of the disease. Mult Scler 21:866-874
4. Jarius S, Eichhorn P, Albert MH, Wagenpfeil S, Wick M, Belohradsky BH, Hohlfeld R, Jenne DE, Voltz R (2007) Intravenous immunoglobulins contain naturally occurring antibodies that mimic antineutrophil cytoplasmic antibodies and activate neutrophils in a TNFalpha-dependent and Fc-receptor-independent way. Blood 109:4376-4382
5. Jarius S, Eichhorn P, Franciotta D, Petereit HF, Akman-Demir G, Wick M, Wildemann B (2017) The MRZ reaction as a highly specific marker of multiple sclerosis: re-evaluation and structured review of the literature. J Neurol 264:453-466
6. Jarius S, Kleiter I, Ruprecht K, Asgari N, Pitarokoili K, Borisow N, Hummert MW, Trebst C, Pache F, Winkelmann A, Beume LA, Ringelstein M, Stich O, Aktas O, Korporal-Kuhnke M, Schwarz A, Lukas C, Haas J, Fechner K, Buttmann M, Bellmann-Strobl J, Zimmermann H, Brandt AU, Franciotta D, Schanda K, Paul F, Reindl M, Wildemann B, in cooperation with the Neuromyelitis Optica Study G (2016) MOG-IgG in NMO and related disorders: a multicenter study of 50 patients. Part 3: Brainstem involvement - frequency, presentation and outcome. J Neuroinflammation 13:281
7. Jarius S, Paul F, Aktas O, Asgari N, Dale RC, de Seze J, Franciotta D, Fujihara K, Jacob A, Kim HJ, Kleiter I, Kumpfel T, Levy M, Palace J, Ruprecht K, Saiz A, Trebst C, Weinshenker BG, Wildemann B (2018) MOG encephalomyelitis: international recommendations on diagnosis and antibody testing. J Neuroinflammation 15:134
8. Jarius S, Paul F, Aktas O, Asgari N, Dale RC, de Seze J, Franciotta D, Fujihara K, Jacob A, Kim HJ, Kleiter I, Kumpfel T, Levy M, Palace J, Ruprecht K, Saiz A, Trebst C, Weinshenker BG, Wildemann B (2018) [MOG encephalomyelitis: international recommendations on diagnosis and antibody testing]. Nervenarzt 89:1388-1399
9. Jarius S, Paul F, Weinshenker BG, Levy M, Kim HJ, Wildemann B (2020) Neuromyelitis optica. Nat Rev Dis Primers 6:85
10. Jarius S, Ruprecht K, Kleiter I, Borisow N, Asgari N, Pitarokoili K, Pache F, Stich O, Beume LA, Hummert MW, Ringelstein M, Trebst C, Winkelmann A, Schwarz A, Buttmann M, Zimmermann H, Kuchling J, Franciotta D, Capobianco M, Siebert E, Lukas C, Korporal-Kuhnke M, Haas J, Fechner K, Brandt AU, Schanda K, Aktas O, Paul F, Reindl M, Wildemann B, in cooperation with the Neuromyelitis Optica Study G (2016) MOG-IgG in NMO and related disorders: a multicenter study of 50 patients. Part 2: Epidemiology, clinical presentation, radiological and laboratory features, treatment responses, and long-term outcome. J Neuroinflammation 13:280
11. Jarius S, Ruprecht K, Kleiter I, Borisow N, Asgari N, Pitarokoili K, Pache F, Stich O, Beume LA, Hummert MW, Trebst C, Ringelstein M, Aktas O, Winkelmann A, Buttmann M, Schwarz A, Zimmermann H, Brandt AU, Franciotta D, Capobianco M, Kuchling J, Haas J, Korporal-Kuhnke M, Lillevang ST, Fechner K, Schanda K, Paul F, Wildemann B, Reindl M, in cooperation with the Neuromyelitis Optica Study G (2016) MOG-IgG in NMO and related disorders: a multicenter study of 50 patients. Part 1: Frequency, syndrome specificity, influence of disease activity, long-term course, association with AQP4-IgG, and origin. J Neuroinflammation 13:279
12. Mao-Draayer Y, Thiel S, Mills EA, Chitnis T, Fabian M, Katz Sand I, Leite MI, Jarius S, Hellwig K (2020) Neuromyelitis optica spectrum disorders and pregnancy: therapeutic considerations. Nat Rev Neurol 16:154-170
13. Probstel AK, Dornmair K, Bittner R, Sperl P, Jenne D, Magalhaes S, Villalobos A, Breithaupt C, Weissert R, Jacob U, Krumbholz M, Kuempfel T, Blaschek A, Stark W, Gartner J, Pohl D, Rostasy K, Weber F, Forne I, Khademi M, Olsson T, Brilot F, Tantsis E, Dale RC, Wekerle H, Hohlfeld R, Banwell B, Bar-Or A, Meinl E, Derfuss T (2011) Antibodies to MOG are transient in childhood acute disseminated encephalomyelitis. Neurology 77:580-588

14. Rinaldi S, Davies A, Fehmi J, Beadnall HN, Wang J, Hardy TA, Barnett MH, Broadley SA, Waters P, Reddel SW, Irani SR, Brilot F, Dale RC, Ramanathan S, Australian, New Zealand MOGSG (2021) Overlapping central and peripheral nervous system syndromes in MOG antibody-associated disorders. Neurol Neuroimmunol Neuroinflamm 8

15. Wildemann B, Jarius S, Franz J, Ruprecht K, Reindl M, Stadelmann C (2021) MOG-expressing teratoma followed by MOG-IgG-positive optic neuritis. Acta Neuropathol 141:127-131
